# Relationship between malnutrition according to the global leadership initiative on malnutrition criteria and oral health among community-dwelling elderly aged 85 years and older: a cross-sectional study

**DOI:** 10.1186/s12903-024-04568-0

**Published:** 2024-08-03

**Authors:** Kensuke Nishio, Takamasa Yoshida, Yasumichi Arai, Tomoka Ito, Shinji Okada, Takayuki Ikeda, Yukiko Abe, Michiyo Takayama, Toshimitsu Iinuma

**Affiliations:** 1 Department of Complete Denture Prosthodontics, Nihon University School of Dentistry, Tokyo, Japan; 2 Center for Supercentenarian Medical Research, Keio University School of Medicine, Tokyo, Japan; 3 Center for Preventive Medicine, Keio University School of Medicine, Tokyo, Japan

**Keywords:** Elderly, GLIM criteria, GOHAI, Malnutrition, Oral health.

## Abstract

**Background:**

A new diagnostic criterion for malnutrition, the Global Leadership Initiative on Malnutrition (GLIM) criteria, has been proposed. Despite a recognized link between malnutrition and oral health, further clarification is needed regarding this association when using the GLIM criteria. This study examined the association between malnutrition and oral health in community-dwelling older adults aged ≥ 85.

**Methods:**

This study was conducted using data from the Tokyo Oldest Old Survey on Total Health study, and altogether 519 participants ≥ 85 years were enrolled. Malnutrition was assessed using the GLIM criteria. Oral health information, on the number of teeth, maximum occlusal force (MOF), saliva production, denture-related questions (dissatisfaction and frequency of use), dental visit history in the past year, whether participants enjoyed meals, and oral-related quality of life was assessed using the Geriatric Oral Health Assessment Index (GOHAI) were collected. MOF was assessed the average values of three measurements and lower tertile by sex as decline in MOF. For GOHAI, the score for each items (Q1-Q12) was also evaluated, and further, the decline in each item (score: 1–2 points on a 5-point scale) was assessed as a “problem with each items.” Oral health factors differing between those with and without malnutrition were analyzed. For differing items, malnutrition risk was evaluated using Cox regression.

**Results:**

Eighty-nine (17.1%) participants experienced malnutrition. Significant differences were observed in the decline in MOF, enjoyment of meals, individual scores for Q2, Q4, and Q6, and the problem with Q3, Q6, Q7, and Q11. Cox regression analysis showed that decline in MOF (odds ratio [OR]: 1.728, 95% confidence interval [CI]: 1.010–2.959), enjoyment of meals (OR: 0.502, 95% CI: 0.289–0.873), problem with Q3 (OR: 5.474, 95% CI: 1.301–23.028), Q6 (OR: 5.325, 95% CI: 1.026–27.636), and Q7 (OR: 2.867, 95% CI: 1.397–5.882) were associated with ORs of malnutrition.

**Conclusion:**

Decline in MOF, enjoyment of meals, swallowing problem (problem with Q3), limit contact due to oral condition (problem with Q6), and esthetics problem (problem with Q7) were associated with malnutrition as assessed using the GLIM criteria.

**Supplementary Information:**

The online version contains supplementary material available at 10.1186/s12903-024-04568-0.

## Background

Currently, the global population is aging. The aging rate is projected to exceed 20% of the global population by the year 2050. This is not the current peak, and further increases in the elderly are expected [1]. Consequently, growing emphasis exists on well-being and how the elderly can live healthier lives [2]. Frailty and sarcopenia are risk factors for healthy longevity [3], and their prevention is essential for the well-being of the elderly [4]. Although several systemic factors are involved in frailty and sarcopenia, nutritional intake may be particularly relevant in dentistry. Protein intake is also associated with the development of frailty [5]. The link between oral function and nutritional status has been reported in several studies [6–8]. However, some of these reports sampled a large number of elderly participants who did not have dentures or other necessary prosthetic treatments for missing teeth, and others collected oral information from the participants’ questionnaires [9]. Therefore, the relevance of these findings needs further detailed investigation.

Furthermore, the lack of consensus on the assessment of nutritional status is a significant problem, although various tools have been used to date [10]. The mini-nutritional assessment (MNA) and MNA-short form (MNA-SF), which are regularly screened for malnutrition, are simple and often used to examine the relationship between oral health and nutritional status [11, 12]. However, these are screening tools for malnutrition and not for malnutrition. Recently, the Global Leadership Initiative on Malnutrition (GLIM) criteria have been published as international standards for diagnosing malnutrition [13]. This diagnostic criterion evaluates malnutrition regarding present symptoms and etiology and is currently being used worldwide. However, evidence of the association between malnutrition and oral health, as diagnosed using the GLIM criteria, is lacking. Some studies have reported an association between the two; however, the participants were nursing home residents, making it difficult to generalize the results.

With the widespread use of the GLIM criteria, a reexamination of the link between oral health and malnutrition is urgently needed. However, some reports suggest that variations in the number of remaining teeth among the elderly do not lead to differences in the incidence of malnutrition, as determined by the GLIM criteria [14]; therefore, reexamining the various oral factors and GLIM criteria is crucial.

Therefore, this study hypothesized that oral health is related to malnutrition, and thus, aimed to investigate the oral health factors associated with malnutrition.

## Methods

### Study design and participants

This study used data from the Tokyo Oldest Old Survey on Total Health (TOOTH) study. All data were collected between March 2008 and November 2009 [15]. A total of 542 individuals who participated in an in-home interview and clinical examination were enrolled in this study [15]. After excluding 11 patients with missing malnutrition data and 12 with no oral health data, 519 participants were analyzed (female, *n* = 297; male, *n* = 222; age, 87.4 ± 2.3 years). The study was conducted after obtaining ethical approval from the Nihon University School of Dentistry (No. 2003– 20, 2008) and Keio University School of Medicine (No. 20070047, 2007). The TOOTH survey was registered with the UMIN Clinical Trial Registry (ID: UMIN000001842). This study was conducted in accordance with the Strengthening the Reporting of Observational Studies in Epidemiology (STROBE) Statement guidelines (Appendix Fig. 1).

### Oral health assessment

The oral health assessment comprised of face-to-face interviews, oral examinations, and a questionnaire regarding oral health by trained dentists [16]. Oral examinations evaluated the number of teeth (0–32), prosthesis design, and maximum occlusal force (MOF). MOF was defined as the maximum biting force during voluntary clenching. The MOF was measured bilaterally in the first molars using an occlusal force meter (GM10; Nagano Keiki, Inc.). In participants wearing dentures, MOF was measured along with their prostheses [17]. The MOF was evaluated in terms of the measured values (average values of three measurements) and the percentage of participants with a decline in the MOF. The decline in MOF was defined as the lower tertile of the MOF for each sex (cut off: male: 10.3 kgf, female: 7.0 kgf). Resting saliva was collected using the 3-minute spit method.

**Fig. 1:**
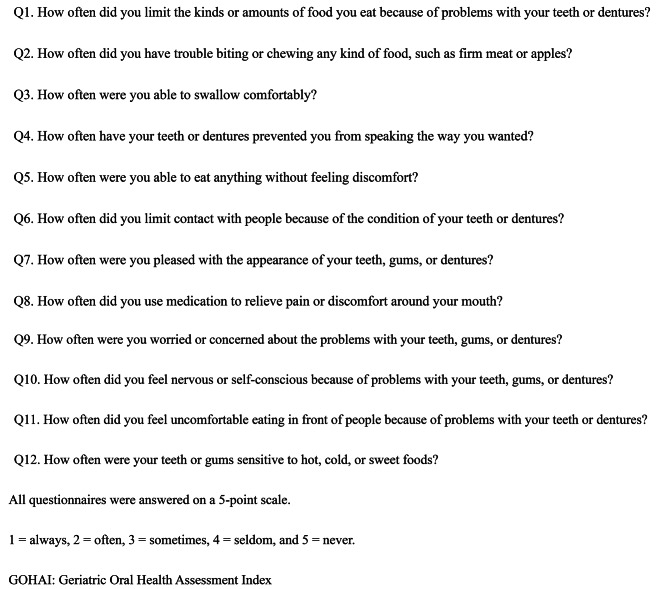
GOHAI questionnaire items

The present study investigated six aspects of oral health based on data obtained from a questionnaire, which included oral health-related quality of life (OHRQoL), two questions about dentures, dental consultation history, and the respondents’ enjoyment of meals. OHRQoL was evaluated using the Geriatric Oral Health Assessment Index (GOHAI) [18], based on a total score of 60 points for 12 questions (Fig. 1). This study used the total score (12–60) and the score for each question item (1–5) to evaluate the GOHAI. Moreover, the percentage of participants with decline in each questions was calculated. Scores of 1 and 2 were considered as problems with each items. The questionnaires were administered regarding dentures, inquiring about the frequency of denture usage and any associated complaints among individuals who wore dentures (Appendix Fig. 2). Both questions were answered on a scale of 5 (1 = always, 2 = often, 3 = sometimes, 4 = seldom, and 5 = never). For the question on frequency, participants who responded with a 1 or 2 were defined as “frequency of use dentures (almost every day).” For the question on the presence or absence of complaints about dentures, participants who answered 1 or 2 were defined as having complaints about dentures. Other questions included, “Do you enjoy meals?” and “Have you visited to the dental clinic within the past year?” The same five-point questionnaire was used to evaluate the question “Do you enjoy meals?”. The participants who scored 1 and 2 were considered to be enjoying their meals (Appendix Fig. 2).

### Demographics and general health assessment

Relevant demographic and general health information including age, sex, household details, education, drinking habits and tobacco smoking, cognitive function, and systemic diseases were collected during face-to-face interviews. Drinking and smoking were dichotomized by whether the participants had ever or had never consumed alcohol and whether they had ever or had never smoked, respectively. Household composition was assessed based on whether the participants lived alone or not. Education level was recorded as a binary variable, indicating whether the participants had graduated from high school. Activities of daily living (ADL) were evaluated using the Barthel Index [19], and disability in ADL was defined as impairment according to one or more indices. Calf circumference was measured twice, and the mean value was used. The World Health Organization Five Well-Being Index (WHO-5) was used to assess psychological status. The participants’ cognitive states were graded using the Mini-Mental State Examination (MMSE) [20], and the percentage of participants with a score less than 24, defined as suspected cognitive impairment [21, 22], was calculated. Physical status was assessed using the timed up-and-go (TUG) test and handgrip strength. To evaluate lower limb function, participants underwent the TUG test [23]. The assessment was conducted by a team of two, primarily a physical therapist and an assistant, to ensure the safety of the participants and prevent any potential falls or injuries during the test. The grip strength was determined as the average value of two measurements. Body mass index (BMI) was calculated from height and weight measurements. Non-fasting blood samples were used, and C-reactive protein (CRP) was measured using standard assay methods. Existing medical conditions were assessed using the International Classification of Diseases, 10th revision. Cardiovascular diseases were recorded, including angina, stroke, myocardial infarction, and transient ischemic attack. Hypertension was defined as the current use of antihypertensive medications or presenting a systolic blood pressure > 140 mmHg during the baseline examination. Considering the high prevalence of undiagnosed diabetes mellitus in the elderly, a confirmed diagnosis was made when at least one of the subsequent criteria was met: (1) self-reported diagnosis, (2) use of insulin or other oral antidiabetic drugs, (3) glycated hemoglobin (HbA1c) ≥ 6.5%, or (4) random blood sugar ≥ 200 mg/dl.

### Definition of malnutrition

Malnutrition was assessed using the GLIM criteria [13]. The evaluation of GLIM criteria consists of two aspects: phenotypic (3 items) and etiology (2 items). The diagnosis requires at least 1 phenotypic and 1 etiologic criterion. In this study, malnutrition was assessed using the following evaluation items: (1) Phenotypic: Non-volitional weight loss: questionnaire results concerning “weight loss of over 3 kg in a year.” Low BMI: <20. Reduced muscle mass: calf circumference (male: <34 cm, female: <33 cm). The cutoff was set based on the Asian consensus [24]. (2) Etiology: Reduction of food intake: GOHAI question 1 (How often do you limit the types or amounts of food you eat because of problems with your oral condition?) [18]. If the answer was “always” or “often”, a reduction in food intake was indicated. Disease burden/inflammation: CRP (> 0.3 mg/dl) [13].

### Statistical analysis

The collected data were imported into SPSS version 26.0 (SPSS Inc., Chicago, IL, USA) for statistical analysis. Descriptive data were summarized as mean with standard deviation (SD) or median with interquartile range (IQR) for continuous variables and frequency (percentages) for categorical variables. The differences between the four categories were assessed using an analysis of variance. The *T*-test and Mann-Whitney *U* test were used to evaluate continuous variables, and the chi-square test was used for categorical variables. Odds ratio (OR) and 95% confidence intervals (CIs) for malnutrition were estimated using Cox regression analysis. The following 8 items were selected for confounding factors: sex (male/female), age (continuous), ADL disability, living alone status, MMSE score (< 24), medical history (cardiovascular disease, cancer) and number of teeth (continuous). All tests were two-sided with a statistical significance level of 5%.

A post-hoc analysis of power showed that the power of multiple regression analysis on the development of malnutrition, the primary outcome of this study, was reasonable at > 0.80 (G*Power 3.1.9.7, effect size: 0.015, total sample size = 519, and number of predictors = 9).

## Results

Eighty-nine (17.1%) participants experienced malnutrition. Baseline characteristics of participants according to malnutrition are listed in Table 1. Accordingly, malnutrition was significantly associated with BMI (*P* = 0.003), calf circumference (*P* < 0.001), weight loss (*P* < 0.001), MMSE score < 24 (*P* = 0.005), TUG test (*P* = 0.016), and CRP (*P* < 0.001). The only items other than the GLIM criteria that showed significant differences were the MMSE score < 24 and the TUG test (Table 1).

**Table 1:** Baseline demographic and general health characteristics of participants according to malnutrition presence or absence

	All	Nomal nultritional status	Malnutiriton	
Varibles	(*n* = 519)	(*n* = 430)	(*n* = 89)	*P value*
Demographics				
Sex (% female)	57.2	57.4	56.2	0.827
Age (y), mean (SD)^a^	87.4 (2.3)	87.3 (2.2)	87.6 (2.4)	0.219
Higher education (%)^b^	24.8	25.7	20.7	0.329
Smoking (%)^b^	38.8	38.4	40.7	0.691
Drinking (%)^c^	47.8	48.4	44.8	0.539
Living alone (%)^d^	34.3	34.1	35.2	0.844
General health assessment				
Body mass index, mean (SD)^ae^	21.5 (3.2)	21.7 (3.2)	20.5 (2.9)	0.003
WHO5, median (IQR)^c^	20 (16–23)	20 (16–23)	18 (14–21)	0.097
ADL disability (%)^f^	25.7	24.7	30.3	0.269
Calf Circumference (cm), median (IQR)^g^	32.2 (30.1–34.4)	32.6 (30.3–34.7)	31.0 (29.8–32.7)	< 0.001
Weight loss (%)^h^	16.2	13.2	30.3	< 0.001
MMSE < 24 (%)^g^	19.9	17.6	30.7	0.005
Physical status				
Handgrip strength (kgf), mean (IQR)^i^	19.3 (15.5–24.0)	19.3 (15.5–24.4)	18.5 (14.5–22.0)	0.098
Timed up & go test (sec), median (IQR)^j^	13.2 (10.9–16.2)	13.0 (10.8–16.0)	14.4 (12.0–16.9)	0.016
Medical history (%)				
Hypertensionk	82.1	82.6	79.8	0.533
Diabetes mellitus	15.8	16.5	12.4	0.328
Cardiovascular disease	21.2	22.1	16.9	0.271
Cancer	18.9	19.1	18.0	0.811
Biochemical				
CRP (mg/dL), median (IQR)	0.09 (0.04–0.17)	0.08 (0.04–0.15)	0.41 (0.11–0.80)	< 0.001

Note: *P-values were calculated for categorical covariates using the chi-squared test, whereas p-values were calculated using the Mann–Whitney U test for continuous variables unless otherwise indicated. ^a^Calculated using parametric *T*-test. ^b–q^Data available for b500, c504, d507, e517, f514, g513, h512, i518, and j445 individuals. SD: standard deviation, WHO-5: World Health Organization Five Well-Being Index, IQR: interquartile range, ADL: activities of daily living, MMSE: Mini-Mental State Examination, CRP: C-reactive protein

The baseline oral health parameters of the participants according to malnutrition are presented in Tables 2 and 3. Malnutrition was significantly associated with male MOF (*P* = 0.019), a decline in MOF (*P* = 0.013), and enjoyment of meals (*P* = 0.002). No significant differences were observed in the number of teeth. The GOHAI results are shown in Table 3. The total GOHAI scores did not show a significant difference. In contrast, individual scores Q2 (trouble biting or chewing, *P* = 0.044), Q4 (unable to speak clearly, *P* = 0.027), and Q6 (limit contacts due to oral condition, *P* = 0.010), problem with Q3 (swallowing problem, *P* = 0.005), problem with Q6 (*P* = 0.032), problem with Q7 (esthetic problem *P* = 0.002), and problem with Q11 (uncomfortable eating in front of others, *P* = 0.029) indicated significant differences. According to the results, six explanatory variables were selected as follow: “decline in MOF”, “enjoyment of meals”, and “problem with Q3, 6,7 and 11”.

**Table 2:** Baseline oral health characteristics of participants according to the presence or absence of malnutrition

	All	Nomal nultritional status	Malnutiriton	
Varibles	(*n* = 519)	(*n* = 430)	(*n* = 89)	*P value*
Number of teeth (continous), median (IQR)^a^	6 (0–17)	7 (0–18)	5 (0–13)	0.270
Number of teeth > 19 (%)^a^	20.0	21.3	13.5	0.093
Maximum Occulusal Force, median (IQR)				
Male^b^	13.8 (7.9–23.7)	15.2 (8.6–24.8)	10.7 (5.9–17.9)	0.019
Female^c^	10.1(5.6–16.5)	10.2 (6.0-16.4)	9.6 (4.2–16.7)	0.333
Decline in MOF (%)^d^	33.3	30.9	45.1	0.013
Enjoyment of meals (%)^e^	81.4	83.6	70.8	0.005
Saliva ml / 3min^f^	1.25 (0.64–1.87)	1.26 (0.65–1.85)	1.12 (0.64–1.93)	0.607
Denture questions (denture wearers only)				
Frequency of use dentures, almost every day (%)^g^	94.1	93.6	96.3	0.346
Having complaints of denture (%)^h^	16.6	15.6	21.0	0.240
Visiting the dental clinic in the past year (%)	59.5	59.5	59.6	0.998

Note: *P-values were calculated for categorical covariates using the chi-square test, whereas *P*-values were calculated

using the Mann–Whitney *U* test for continuous variables

^a–i^Data available for a516, b213, c270, d483, e517, f427, g454, and h446 individuals. IQR: interquartile range, MOF: maximum occlusal force

**Table 3:** Baseline GOHAI scores of participants according to the presence or absence of malnutrition

	All	Nomal nultritional status	Malnutiriton	
Varibles	(*n* = 519)	(*n* = 430)	(*n* = 89)	*P value*
Total GOHAI, median (IQR)	56 (51–58)	56 (51–58)	54 (50–58)	0.225
Individual questions				
Q1: limit the kinds of food, median (IQR)	5 (3–5)	5 (3–5)	5(3–5)	0.940
Problem with Q1 (%)	17.1	16.4	22.2	0.254
Q2: trouble biting or chewing, median (IQR)^a^	4 (3–5)	4 (3–5)	3 (2–5)	0.044
Problem with Q2 (%)	16.8	16.1	19.5	0.396
Q3: able to swallow comfortably, median (IQR)	5 (5–5)	5 (5–5)	5 (5–5)	0.396
Problem with Q3 (%)	1.9	1.2	5.0	0.005
Q4:unable to speak clearly, median (IQR)	5 (4–5)	5 (4–5)	5 (4–5)	0.027
Problem with Q4 (%)	5.8	4.9	10.1	0.054
Q5: able to eat without discomfort median (IQR)^a^	5 (4–5)	5 (4–5)	5 (3–5)	0.436
Problem with Q5 (%)	7.2	6.5	10.2	0.220
Q6: limit contacts with people, median (IQR)	5 (5–5)	5 (5–5)	5 (5–5)	0.010
Problem with Q6 (%)	1.2	0.7	3.4	0.032
Q7: pleased with look of teeth (IQR)^b^	5 (5–5)	5 (5–5)	5 (5–5)	0.485
Problem with Q7 (%)	7.7	6.1	15.7	0.002
Q8: used medication to relieve pain, median (IQR)	5 (5–5)	5 (5–5)	5 (5–5)	0.519
Problem with Q8 (%)	0.8	0.9	0	0.470
Q9: worried about teeth, gums or dentures, median (IQR)^c^	5 (4–5)	5 (4–5)	5 (3–5)	0.281
Problem with Q9 (%)	8.1	8.0	9.0	0.747
Q10: Self-onscious of teeth, gums or dentures, median (IQR)^b^	5 (5–5)	5 (5–5)	5 (5–5)	0.752
Problem with Q10 (%)	2.9	2.8	3.4	0.769
Q11: Uncomfortable eating in front of others, median (IQR)	5 (5–5)	5 (5–5)	5 (5–5)	0.874
Problem with Q11 (%)	2.9	2.1	6.7	0.029
Q12: Sensitive to hot, cold or sweet foods, median (IQR)^b^	5 (5–5)	5 (5–5)	5 (5–5)	0.767
Problem with Q12 (%)	1.7	1.9	1.1	0.524

Note: *P-values were calculated for categorical covariates using the chi-square test, and for continuous variables using the Mann–Whitney *U* test

^a–d^Data available for a517, b518, and c516 individuals. GOHAI: Geriatric Oral Health Assessment Index, IQR: interquartile range

The results of the Cox regression analysis are presented in Table 4. Oral health items associated with malnutrition were used as dependent variables to evaluate the ORs. Decline in MOF (OR: 1.728, 95% CI: 1.010–2.959), enjoyment of meals (OR: 0.502, 95% CI: 0.287–0.868), problem with Q3 (OR: 5.474, 95% CI: 1.301–23.028), problem with Q6 (OR: 5.325, 95% CI: 1.026–27.636), and problem with Q7 (OR: 2.867, 95% CI: 1.397–5.882) indicated significant differences after adjusting for all confounding factors.

**Table 4:** Odds ratio of malnutrition for each oral health item

		Model 1				Model 2	
		OR (95% CI)	*P* value			OR	*P* value
Decline of MOF		1.837 (1.132–2.980)	0.014			1.728 (1.010–2.959)	0.046
Enjoyment of meals		0.474 (0.281-0.800)	0.003			0.502 (0.289–0.873)	0.015
Problem with Q3		5.060 (1.433–17.864)	0.012			5.474 (1.301–23.028)	0.020
Problem with Q6		4.965 (0.986–25.014)	0.052			5.325 (1.026–27.636)	0.047
Problem with Q7		2.893 (1.444–5.797)	0.003			2.867 (1.397–5.882)	0.004
Problem with Q11		3.382 (1.172–9.755)	0.024			2.990 (0.987–9.060)	0.053

Model 1: Unadjusted model

Model 2: Adjusted for age and sex. ADL disability, living alone, MMSE score (< 24), medical history (cardiovascular disease or cancer), and number of teeth (continuous)

OR: odds ratio, CI: confidence interval, MOF: maximum occlusal force, GOHAI: Geriatric Oral Health Assessment Index, ADL: activities of daily living, MMSE: Mini-Mental State Examination

## Discussion

The assessment of malnutrition varies across countries and regions. The GLIM criteria were developed to establish universal evaluation standards and achieve early detection and early treatment of malnutrition [13]. This study contributes to the fulfillment of evidence linking malnutrition, as assessed by the GLIM criteria, and oral health, reaffirming the importance of maintaining oral health.

In this study, the number of teeth was not associated with malnutrition, whereas decline in MOF and enjoyment of meals were associated with malnutrition. Furthermore, the OHRQoL (as assessed by the GOHAI) was not associated with malnutrition. From the each GOHAI items, swallowing problems (problem with Q3), limit contact due to oral condition (problem with Q6), and esthetic problems (problem with Q7) was associated with malnutrition.

Many previous studies on the association between oral health and malnutrition have used the MNA to assess malnutrition. Hussein et al. meta-analyzed the association between oral function and MNA (including MNA-SF) [25]. The results indicated that a lack of awareness about oral care, regular dentist visits, and the use of dentures in edentulous individuals were associated with the risk of malnutrition. Although this review did not include items that assessed functions, such as occlusal force, and a few items that set oral health, it indicated oral health items associated with the MNA. However, the MNA is only a screening tool for low nutritional status. Ohta et al. evaluated the oral function and nutritional status of hospitalized nonacute patients aged > 70 years [26]. In their study, they assessed the nutritional status using the MNA-SF and GLIM criteria, and the correlation between these two nutritional indices and oral function was analyzed. Interestingly, the MNA-SF scores were correlated with oral function; however, the GLIM criteria showed no correlation. The MNA-SF is a screening tool that carries the risk of false-positive results. A previous study analyzing the MNA-SF scores of subjects diagnosed with malnutrition using the GLIM criteria reported a sensitivity of approximately 60% and a specificity of 80% [27]. Therefore, to analyze the association between oral health, evaluating the relationship between the GLIM criteria and oral health is essential, rather than relying on screening tools.

The GLIM criteria were published in 2018, and only a few studies have reported their relevance to oral health. Ohta et al. examined the association between oral function and GLIM criteria; however, these participants were hospitalized, and many biases must be considered when generalizing their results [26]. In the TOOTH study, the participants visited the measurement site to obtain baseline data. Thus, the data from the TOOTH study were obtained from healthy older adults, and the GLIM Criteria and oral health evidence obtained in this study are valuable. Besides the “number of remaining teeth,” which has been used in previous studies on oral health and nutrition, the study also evaluated items related to “bite strength,” “oral quality of life,” and “dentures,” which may lead to new findings on oral health and malnutrition.

In this study, no significant differences were found in the number of teeth between the malnourished and healthy participants. A meta-analysis of the association between oral health and the MNA [25] found that edentulous patients without further dentures were at risk of malnutrition. This suggests that the number of teeth was not a direct risk factor for malnutrition. Nishio et al. reported that malnutrition affected life expectancy independent of the number of teeth [14]. The results of this study are consistent with these findings. However, a greater proportion of malnourished participants had decreased occlusal force, indicating that occlusal force was a key factor for malnutrition. Iinuma et al. reported that decreased occlusal force was related to life prognosis [17], and the maintenance of occlusal force was an important factor for healthy longevity in the elderly. Although this study did not examine differences in nutrient intake status, the authors consider previous reports [28, 29] that differences in occlusal force affected the food available for intake, resulting in malnutrition. Although tooth loss leads to decreased occlusal force, appropriate prosthetic treatment may help maintain the occlusal force to a certain extent, even if the number of remaining teeth is small [30]. Inomata et al. investigated the relationship between nutrient intake and the number of remaining teeth and occlusal force in older adults. They reported that only occlusal force was associated with nutrient [31]. Assessing the number of remaining teeth is simple and does not require the use of any special equipment. However, when evaluating the impact of the number of remaining teeth on nutritional intake, it is necessary to consider factors such as the periodontal condition and the quality of prostheses. Further investigation into the underlying mechanisms is necessary to better understand this phenomenon. The number of teeth may not be an absolute factor in health in current gerodontology as long as dentures and other treatments are correctly applied to the missing teeth areas.

The total OHRQoL scores measured by the GOHAI did not differ between malnutrition and normal nutritional status participants. OHRQoL and the risk of malnutrition, as assessed by the MNA, have been reported to be associated [32–34]. However, no reports have been published on GLIM criteria-assessed malnutrition and OHRQoL. This study found no difference in the overall GOHAI scores of malnutrition and healthy participants. Sobrini et al. reported a higher probability of being classified as malnutrition when assessed with the MNA-SF than with the GLIM criteria [35]. However, the authors analyzed 12 oral-related items from the GOHAI to obtain further information from each item and found that, problem with Q3 (swallowing problem), problem with Q6 (limit contacts due to oral condition), and problem with Q 7(esthetics problem) was associated with an OR of malnutrition. Several reports exist on the assessment of malnutrition using the GLIM criteria because it relates to swallowing function as opposed to the number of teeth or bite force [36, 37]. Despite being focused on inpatient participants and involving different conditions from those in our study, the essential role of swallowing in nutritional intake makes it reasonable to expect comparable results. However, no reports exist of esthetic problems related to malnutrition. Esthetic problems have been reported as reasons for discontinuing the use of dentures [38]. Discontinuation of dentures for esthetic reasons may have resulted in a decline in masticatory function, leading to a risk of malnutrition. GOHAI questions were categorized into physical functioning, psychosocial functioning, pain, and discomfort. The problem with Q6 (limit contacts due to oral condition) is a psychosocial functioning question. A decline in psychosocial function has been reported as a risk factor for malnutrition [39, 40]. This study showed no association between the presence of malnutrition and the rate of living alone, suggesting that even those who live alone can maintain a certain level of socialization by meeting acquaintances and family members for meals and receiving social services. However, if deterioration of oral health is the reason for the loss of social interaction, the risk of malnutrition may increase, as reported in the literature. Poor oral condition to the point of refraining from being with people is a problem that should not be overlooked by the elderly.

Finally, an interesting result was that participants who enjoyed their meals were less likely to be malnourished. Enjoyment of meals is one factor associated with food intake in the elderly [41]. The reason for not enjoying meals may be due to medical problems, such as systemic illness, or because they live alone. However, in this study, food enjoyment was independently associated with malnutrition even after controlling for confounding factors. Compared to other oral health items, “enjoy eating” may not be a direct oral health item; however, maintaining oral function is linked to enjoyment of meals [42], and this question may be a comprehensive oral health assessment.

This study revealed an association between the onset of malnutrition and oral health according to the GLIM criteria. However, this study has several clinical limitations. First, there are concerns regarding the evaluation methods of the GLIM criteria adopted in this study. “weight loss” was assessed by asking whether the patient had lost 3 kg or more in the past year in the TOOTH study. The definition of “weight loss” in the GLIM criteria is as follows: (1) 5% > within 6 M, (2) 10% > beyond 6 M. The average weight of the participants was 49.7 ± 9.5 kg, so if this weight loss occurred within 6 m, it corresponds to a “weight loss” of 5% or more. However, the questions in this study could not be answered when weight loss occurred. Therefore, the authors’ “weight loss” assessment may have included participants with suspected positive results. The GOHAI questionnaire was used to determine reduced food intake; therefore, digestive symptoms were not considered in this study. Second, the participants were elderly individuals living in a limited area of Tokyo. Comparisons with other areas were impossible; therefore, oral health could not be considered due to regional differences. Third, no items objectively assessed the denture quality. Examples of objective evaluations include evaluating the masticatory function using gummy jellies and assessing the fit of denture bases using fit test materials. An objective assessment of denture quality may provide new insights into the hypothesis that properly functioning dentures with few remaining teeth can prevent malnutrition. Finally, this study is a cross-sectional investigation, which suggests a relationship between oral health and malnutrition but cannot clarify a causal relationship. To strengthen the evidence from this study, future longitudinal investigations are essential. However, evidence examining the association between malnutrition diagnosed by GLIM criteria and oral health is limited, and this study may provide important evidence. Previous studies on this topic have used screening tools such as MNA. In this study, international diagnostic criteria were used to provide valuable evidence regarding the association between oral health and nutrition. Given that the association between malnutrition and oral health has not been examined based on GLIM criteria in healthy older adults, this study fills in the relevant gaps in knowledge and reiterates the importance of oral health in the elderly.

## Conclusions

This study identified the oral health factors associated with the development of malnutrition diagnosed using the GLIM criteria. A decline in MOF increases the risk of malnutrition. Enjoyment of meals also reduces the risk of malnutrition. The overall OHRQoL score assessed using the GOHAI was not associated with malnutrition. However, for each individual question in the GOHAI, problem with swallowing, limit contact due to oral condition, and problem with esthetic were associated with malnutrition.

Oral health related to malnutrition was associated with oral function, enjoyment of meals, and sociability related to oral condition. These results suggest that while maintenance of oral function is important for improving nutrition in the elderly, comprehensive nutrition improvement programs are also needed.

### Electronic supplementary material

Below is the link to the electronic supplementary material.

Supplementary Material 1

## Data Availability

The datasets generated and/or analyzed during the current study are not publicly available in order to protect the participants’ privacy but are available from the corresponding author on reasonable request.

## Abbreviations

MNA: Mini nutritional assessment
MNA-SF: Mini nutritional assessment-short form
GLIM: Global Leadership Initiative on Malnutrition
TOOTH: Tokyo Oldest Old Survey on Total Health
MOF: Maximum occlusal force
OHRQoL: Oral health-related quality of life
GOHAI: Geriatric Oral Health Assessment Index
ADL: Activities of daily living
MMSE: Mini-Mental State Examination
BMI: Body mass index
WHO-5: World Health Organization Five Well-Being Index
SD: Standard deviation
IQR: Interquartile range
OR: Odds ratio
CI: Confidence interval
