# Supplementary material for: Relationship between malnutrition according to the global leadership initiative on malnutrition criteria and oral health among community-dwelling elderly aged 85 years and older: a cross-sectional study
Source: BMC Oral Health. 2024 Aug 3;24:887. doi: 10.1186/s12903-024-04568-0 (PMC11297638; doi:10.1186/s12903-024-04568-0)
Supplement: Supplementary file 1 — Supplementary Material 1 [file 12903_2024_4568_MOESM1_ESM.doc]

Appendix fig. 1: STROBE Statement—checklist of items that should be included in reports of observational studies

|  | Item No | Recommendation |
| --- | --- | --- |
| **Title and abstract** | 1 | (*a*) Indicate the study’s design with a commonly used term in the title or the abstract  (lines 1–3) |
|  |  | (*b*) Provide in the abstract an informative and balanced summary of what was done and what was found (lines22–53) |
| Introduction | | |
| Background/rationale | 2 | Explain the scientific background and rationale for the investigation being reported  (lines58–89) |
| Objectives | 3 | State specific objectives, including any prespecified hypotheses (lines90–91) |
| Methods | | |
| Study design | 4 | Present key elements of study design early in the paper (lines 95–96) |
| Setting | 5 | Describe the setting, locations, and relevant dates, including periods of recruitment, exposure, follow-up, and data collection (line 96) |
| Participants | 6 | (*a*) *Cohort study*—Give the eligibility criteria, and the sources and methods of selection of participants. Describe methods of follow-up (lines 96–100.  The summary of TOOTH study was reported by ref 15). |
|  |  | (*b*) *Cohort study*—For matched studies, give matching criteria and number of exposed and unexposed (lines 202) |
| Variables | 7 | Clearly define all outcomes, exposures, predictors, potential confounders, and effect modifiers. Give diagnostic criteria, if applicable (lines 109–182 and 191–194) |
| Data sources/ measurement | 8* | For each variable of interest, give sources of data and details of methods of assessment (measurement). Describe comparability of assessment methods if there is more than one group (lines 95–182) |
| Bias | 9 | Describe any efforts to address potential sources of bias (The population were randomly drawn from the basic registry of residents of each ward [ref 15]. ) |
| Study size | 10 | Explain how the study size was arrived at (lines 196–199) |
| Quantitative variables | 11 | Explain how quantitative variables were handled in the analyses. If applicable, describe which groupings were chosen and why (lines 184–190) |
| Statistical methods | 12 | (*a*) Describe all statistical methods, including those used to control for confounding  (lines 184–199) |
|  |  | (*b*) Describe any methods used to examine subgroups and interactions N/A |
|  |  | (*c*) Explain how missing data were addressed (lines 98–100) |
|  |  | (*d*) *Cohort study*—If applicable, explain how loss to follow-up was addressed  N/A, cross-sectional study  *Case-control study*—If applicable, explain how matching of cases and controls was addressed N/A  *Cross-sectional study*—If applicable, describe analytical methods taking account of sampling strategy /N/A |
|  |  | (*e*) Describe any sensitivity analyses (lines 196–199) |

Continued on next page

| Results | | |
| --- | --- | --- |
| Participants | 13* | (a) Report numbers of individuals at each stage of study—eg numbers potentially eligible, examined for eligibility, confirmed eligible, included in the study, completing follow-up, and analysed (According to a previous publication (ref 15) |
|  |  | (b) Give reasons for non-participation at each stage (According to a previous publication (ref 15) |
|  |  | (c) Consider use of a flow diagram (According to a previous publication (ref 15) |
| Descriptive data | 14* | (a) Give characteristics of study participants (eg demographic, clinical, social) and information on exposures and potential confounders (lines 202–207) |
|  |  | (b) Indicate number of participants with missing data for each variable of interest (Table 1) |
|  |  | (c) *Cohort study*—Summarise follow-up time (eg, average and total amount) N/A, cross-sectional study |
| Outcome data | 15* | *Cohort study*—Report numbers of outcome events or summary measures over time  (lines 202–203) |
|  |  | *Case-control study—*Report numbers in each exposure category, or summary measures of exposure N/A |
|  |  | *Cross-sectional study—*Report numbers of outcome events or summary measures (lines 202–203) |
| Main results | 16 | (*a*) Give unadjusted estimates and, if applicable, confounder-adjusted estimates and their precision (eg, 95% confidence interval). Make clear which confounders were adjusted for and why they were included (lines 191–199, lines 220–225, and table 4) |
|  |  | (*b*) Report category boundaries when continuous variables were categorized (lines 114–117) |
|  |  | (*c*) If relevant, consider translating estimates of relative risk into absolute risk for a meaningful time period N/A, cross-sectional study |
| Other analyses | 17 | Report other analyses done—eg analyses of subgroups and interactions, and sensitivity analyses NA |
| Discussion | | |
| Key results | 18 | Summarise key results with reference to study objectives (lines233–238) |
| Limitations | 19 | Discuss limitations of the study, taking into account sources of potential bias or imprecision. Discuss both direction and magnitude of any potential bias (lines 328–347) |
| Interpretation | 20 | Give a cautious overall interpretation of results considering objectives, limitations, multiplicity of analyses, results from similar studies, and other relevant evidence (lines 347–354) |
| Generalisability | 21 | Discuss the generalisability (external validity) of the study results(lines 338–340) |
| Other information | | |
| Funding | 22 | Give the source of funding and the role of the funders for the present study and, if applicable, for the original study on which the present article is based (lines 400–407) |

*Give information separately for cases and controls in case-control studies and, if applicable, for exposed and unexposed groups in cohort and cross-sectional studies.

**Note:** An Explanation and Elaboration article discusses each checklist item and gives methodological background and published examples of transparent reporting. The STROBE checklist is best used in conjunction with this article (freely available on the Web sites of PLoS Medicine at http://www.plosmedicine.org/, Annals of Internal Medicine at http://www.annals.org/, and Epidemiology at http://www.epidem.com/). Information on the STROBE Initiative is available at [www.strobe-statement.org](http://www.strobe-statement.org/).

Appendix fig. 2: 5-point scale oral health questionnaire

Denture questions

1) Frequency of use dentures

Do you use your dentures?

2) Having complaints of denture

　 Do you have complaints about your dentures?"

Enjoyment of meals

Do you enjoy meals?

All questions were answered as follows:

1 = always, 2 = often, 3 = sometimes, 4 = seldom, and 5 = never.
